# Endocytosis and Lack of Cytotoxicity of Alkyl-Capped Silicon Quantum Dots Prepared from Porous Silicon

**DOI:** 10.3390/ma12101702

**Published:** 2019-05-25

**Authors:** Wipaporn Phatvej, Harish K. Datta, Simon C. Wilkinson, Elaine Mutch, Ann K. Daly, Benjamin R. Horrocks

**Affiliations:** 1 Thailand Institute of Scientific and Technological Research, Bangkok 10900, Thailand; nuuwipaporn@hotmail.com; 2 The James Cook University Hospital, Marton Road, Middlesbrough TS4 3BW, UK; harish.datta@nuth.nhs.uk; 3 Institute of Cellular Medicine, Medical School, Newcastle University, Newcastle NE1 7RU, UK; simon.wilkinson@newcastle.ac.uk (S.C.W.); a.k.daly@newcastle.ac.uk (A.K.D.); 4 Toxicology Unit, Medical School, Newcastle University, Newcastle NE1 7RU, UK; elaine.mutch@yahoo.com; 5 Chemical Nanoscience Laboratory, School of Natural and Environmental Sciences, Newcastle University, Newcastle NE1 7RU, UK

**Keywords:** porous silicon, quantum dot, endocytosis, cytotoxicity, caveolin, clathrin, cholesterol

## Abstract

Freely-dissolved silicon quantum dots were prepared by thermal hydrosilation of 1-undecene at high-porosity porous silicon under reflux in toluene. This reaction produces a suspension of alkyl-capped silicon quantum dots (alkyl SiQDs) with bright orange luminescence, a core Si nanocrystal diameter of about 2.5 nm and a total particle diameter of about 5 nm. Previous work has shown that these particles are rapidly endocytosed by malignant cell lines and have little or no acute toxicity as judged by the standard 3-(4,5-dimethylthiazol-2-yl)-2,5-diphenyltetrazolium bromide (MTT) assay for viability and the Terminal deoxynucleotidyl transferase dUTP nick end labeling (TUNEL) assay for apoptosis. We have extended this work to the CACO-2 cell line, an established model for the human small intestinal mucosa, and demonstrate that neither acute nor chronic (14 days) toxicity is observed as judged by cell morphology, viability, ATP production, ROS production and DNA damage (single cell gel electrophoresis) at doses of 50–200 μg mL${}^{-1}$. Quantitative assessment of the extent of uptake of alkyl SiQDs by CACO-2, HeLa, HepG2, and Huh7 cell lines by flow cytometry showed a wide variation. The liver cell lines (HepG2 and Huh7) were the most active and HeLa and CACO-2 showed comparable activity. Previous work has reported a cholesterol-sensitivity of the endocytosis (HeLa), which suggests a caveolin-mediated pathway. However, gene expression analysis by quantitative real–time polymerase chain reaction (RT-PCR) indicates very low levels of caveolins 1 and 2 in HepG2 and much higher levels in HeLa. The data suggest that the mechanism of endocytosis of the alkyl SiQDs is cell-line dependent.

## 1. Introduction

Quantum dots are now as well-established as luminescent labels in cell biology [1,2]. Their advantages over molecular dyes include: resistance to photofading, broad absorption spectra and narrow, bright photoluminescence spectra [3]. However, they suffer from two important limitations. They are unsuitable for in-vivo use, because they contain heavy metals which may, over long durations, leach from the particles [4,5]. They are also large, complex nanoscale objects because of the necessity to cap the luminescent core with a large band gap semiconductor shell to maintain high quantum yields and then to coat the shell with a polymer that facilitates conjugation of the quantum dot to antibodies or nucleic acids for targeting. The large hydrodynamic radius of the whole object may restrict access to parts of the cell or tissue under study and even perturb the behavior of the molecule is it used to label [6]. Silicon quantum dots (SiQDs) are now intensively investigated because of their promise as non-toxic luminescent labels in life science applications [7]. Studies on related silicon nanomaterials such as porous silicon nanoparticles suggest that the silicon is converted to orthosilicate and removed by renal clearance without obvious cytotoxicity [8]. Porous silicon has now been applied successfully in biomedicine [9]. In addition, nanoscale silicon also possesses a rich surface chemistry, in many respects analogous to molecular hydrosilanes [10], that facilitates direct conjugation of organic molecules to the luminescent core. Finally, SiQDs emit in the red/near infrared region of the spectrum at small sizes (diameters 2–3 nm) [11].

**Figure 1:**
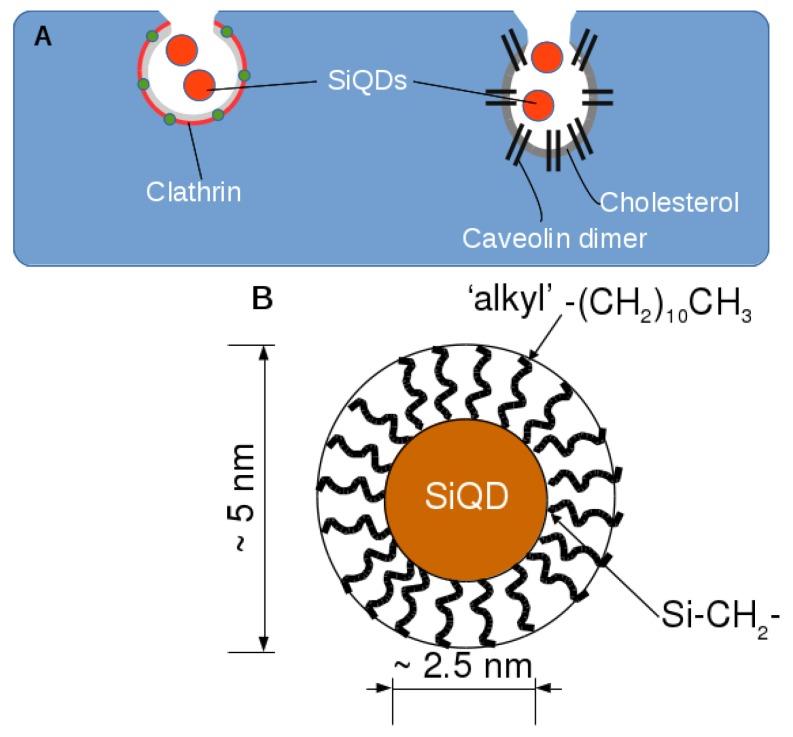
(**A**) Simplified schematic of the initial stages of two selected mechanisms of endocytosis. On the left, clathrin-mediated endocytosis and on the right, cholesterol-dependent endocytosis in caveolae. The orange discs represent SiQDs undergoing endocytosis; their size is exaggerated compared to the clathrin/caveolin coated pit dimensions of 50–100 nm. (**B**) Schematic of an alkyl SiQD. The luminescent QD core is crystalline Si of approximate diameter 2.5 nm. The QD is capped by n-alky chains anchored to the surface of the core by Si-C bonds. The total diameter of the particle is about 5 nm.

There is a range of methods to prepare silicon quantum dots [6,7], which principally divide into molecular routes in which Si compounds are transformed to elemental Si, and methods that involve etching of elemental Si to reduce the size of the particles below the threshold for quantum confinement. Some successful procedures involve a combination of chemical reaction and etching [12,13]. We prepare alkyl-capped silicon quantum dots (alkyl SiQDs) by hydrosilation of 1-undecene at porous silicon layers prepared from low-doped Si, etched at high current density near the threshold for electropolishing [14,15]. These quantum dots are stable to aqueous environments for periods of several days [16]. They show orange photoluminescence and have quantum yields of about 10–20%. The obvious disadvantages of this preparation method are the low quantity of material produced, typically 200 μg from a 1 cm${}^{2}$ Si chip, and the hazardous nature of the aqueous hydrogen fluoride (HF) etch. The major advantages are that the process starts from pure Si wafer and these alkyl SiQDs have been extensively characterized in our laboratories with respect to size, structure [15], chemical stability [16], photophysics [17,18,19] and their fluorescence intensity provides a quantitative measure of concentration [16]. Figure 1B illustrates the structure and dimensions of these alkyl SiQDs, which are used throughout this report.

Cytotoxicity evaluation of these alkyl SiQDs has been carried out in HeLa (immortalized epithelial cervical carcinoma) and SW1353 cell lines (chondrosarcoma) [20]. No adverse effects were observed on HeLa and SW1353 in terms of cell morphological features (membrane sinking or blebbing), cell viability, proliferation or cell apoptosis assays. However, it is known that surface charge has a strong effect on the internalization and cytotoxicity of nanoparticles [21]. Studies on SiQDs with different organic capping layers, have found a dependence on the surface chemistry. SiQDs bearing cationic monolayers showed more cytotoxicity and induced higher oxidative stress compared with neutral alkyl monolayers, while anionic monolayers showed very low cytotoxicity effects in macrophage NR8383 cells [22]. SiQDs bearing reactive epoxide functional groups were observed to show greater cytotoxicity than the corresponding diols [23]. Liver cell lines (HHL-5, HepG2) and embryonic cells (3T3-L1) treated with SiQDs capped with poly-acrylic acid (PAAc) revealed no adverse effects in cell morphology, cell proliferation, viability, and DNA damage assays [24]. Evidence of the influence of dopant on bacterial toxicity of SiQDs has been reported recently [25]. The inflammatory cellular response of nanoparticles has been studied by looking for cytotoxicity in phagocytic cells [26]. Macrophage cells (RAW 264.7) were treated with two types of silicon particles with diameter 3 nm (nanodots/SiQDs) and 100–300 nm (microdots) and some effects on the cellular inflammatory responses were found. The SiQDs were associated with decreased synthesis of tumour necrosis factors-alpha (TNF-α) and interleukin 6 (IL-6)). Microdots were less toxic to macrophages, but induced increased production of IL-6 and TNF-α at relatively low concentrations [27]. A pharmacokinetic assessment using an in vivo model is also important to determine the toxicity effects on distribution, clearance and specific organs [28,29]. Nevertheless, the mechanism of endocytosis of SiQDs and their possible chronic toxicity is not yet well understood.

This paper investigates the toxicity and the mechanism of internalization of silicon quantum dots in human cell lines. ${}^{n}C_{11}$-alkyl-capped silicon quantum dots (alkyl SiQDs) were chosen for this investigation because their physical properties have been well-characterized. The CACO-2 cell line was chosen for a detailed investigation of the cytotoxicity because of its relevance as a model for the effects of the ingestion of nanoparticles. The other cell lines (HeLa, HepG2 and Huh7) were chosen to probe the nature of the internalization—previous work on HeLa indicated a cholesterol sensitivity of the process [20]. As will be demonstrated below, the other cell lines have widely varying amounts of caveolins. Figure 1 illustrates schematically two important types of endocytosis [30].

## 2. Results and Discussion

**Figure 2:**
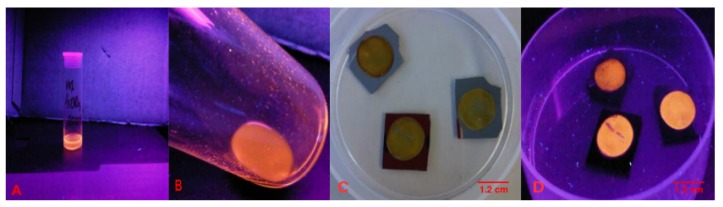
(**A**) ${}^{n}C_{11}$ alkyl-capped SiQDs as a toluene sol and (**B**) after evaporation of the toluene; both photographs taken under illumination with an Hg lamp (λ=365 nm) to show the orange fluorescence. (**C**) The porous silicon, an approximately circular, brown–yellow area formed on the top of the silicon chip. (**D**) A fluorescent image of porous silicon under UV-lamp (Hg, λ=365 nm).

Typical preparations of alkyl SiQDs are shown in Figure 2. The porous silicon layer appears as a circular brown/yellow disc on the 1 cm${}^{2}$ Si chips after the anodic etch, Figure 2C. Under illumination with a handheld Hg lamp, the porous silicon disc emits strong orange photoluminescence (Figure 2D). After reflux in a toluene solution of 1-undecene, the porous silicon layer appears to dissolve and produces a transparent, yellow sol which also emits orange light under the Hg lamp. The sol may be evaporated to dryness under vacuum and the alkyl SiQDs easily redisperse in nonpolar solvents. Owing to the hydrophobic nature of the alkyl capping monolayer the particles do not disperse directly in aqueous media. However, they do form a kinetically-stable lyophobic sol upon injection of a dispersion in organic solvent into water [16]. In order to prepare dispersions of alkyl SiQDs in culture media for the cytotoxicity and endocytosis experiments, we prefer to use diethyl ether as the solvent. The advantage of ether as a vehicle for dispersion of alkyl SiQDs is that its high volatility results in minimal contamination of the culture medium with organic solvent. However, in the experiments reported below, we do control for the possible cytotoxicity of residues of the vehicle in the absence of SiQDs.

### 2.1. Cytotoxicity

**Figure 3:**
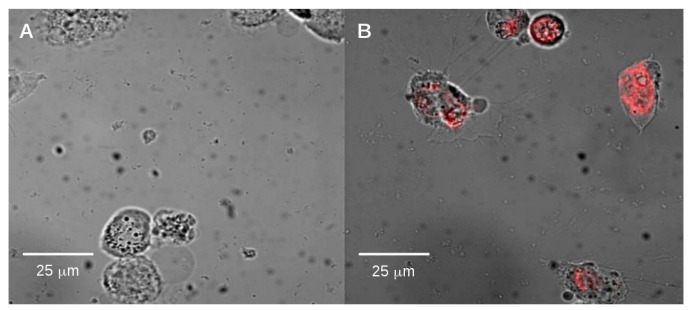
(**A**) CACO-2 cells treated for 4 h with blank vehicle (0.1% *v*/*v* diethyl ether) and (**B**) with vehicle + SiQDs (0.2 μg mL${}^{-1}$). The (orange–red) false color fluorescent image is superimposed on the bright field optical images indicating internalization of SiQDs in CACO-2.

First we examined the internalization of alkyl-SiQDs by CACO-2 cells. The CACO-2 cell line is derived from a large intestine carcinoma, but in culture may resemble the enterocytes of the small intestine [31]. CACO-2 cells are widely used as an in vitro model of human small intestine mucosa [32] and are therefore relevant for assessment of the effects of ingestion of nanoparticles. Figure 3 shows a representative epifluorescence image (false color) at low alkyl-SiQD concentration overlaid on the optical image of the cells. These images and the flow cytometry data (below, Figure 11) confirm the internalization of alkyl-SiQDs by CACO-2.

**Figure 4:**
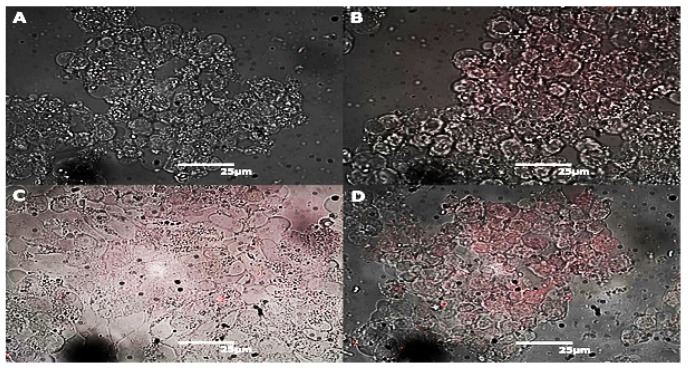
Optical images of CACO-2 cells exposed to alkyl-SiQDs. (**A**) The bright field image shows the morphology of CACO-2 in the control group (0.1% *v*/*v* ether), and images (**B**–**D**) show the morphology of 14 day-treated CACO-2 with SiQDs (0.5, 5, or 50 μg mL${}^{-1}$) respectively. The fluorescence of the alkyl-SiQDs is overlaid on images (**B**–**D**) in false color and the brightness and contrast have been adjusted.

Next, we observed the morphology of the CACO-2 cells upon longer-term exposure to alkyl-SiQDs. Cells were observed at time points of (0.5, 1, 2, 4, 24 h or 14 days) and at alkyl-SiQDs concentrations of (0, 0.2, 0.5, 5.0, or 50 μg mL${}^{-1}$). The integration time of the CCD was automatically adjusted by the instrument software and we used these images for a qualitative comparison only; quantitative data was obtained from flow cytometry below. Figure 4 shows the morphology of CACO-2 cultured in the presence of up to 50 μg mL${}^{-1}$ alkyl-SiQDs for 14 days.

No change in morphology was evident upon exposure of CACO-2 to alkyl-SiQDs compared to the control and the fluorescence of the quantum dots was clearly visible and associated with the cells. This is clearest in Figure 4D from the absence of fluorescence in the cell-free areas of the image. Some aggregation of alkyl-SiQDs was observed as more intense red spots at the higher concentrations (Figure 4C,D); this was not surprising given the insolubility of alkyl-SiQDs in aqueous media.

In previous work, a lack of acute toxicity of alkyl SiQDs in HeLa and SW1353 cells has been observed [20]. Those investigations considered viability, proliferation and apoptosis for relatively low exposures of alkyl-SiQDs for times up to 24 h. Amine-terminated SiQDs of mean core diameter 4.6 nm were also found to lack significant adverse effect on HepG2 cell viability by the 3-(4,5-dimethylthiazol-2-yl)-2,5-diphenyltetrazolium bromide (MTT) assay [33]. A lack of cytotoxicity has also been reported for laser-synthesized SiQDs [34]. Other workers have shown some cytotoxicity of biogenic Si/SiO${}_{2}$ particles in the A431 human epithelial cell line after 3 h of exposure to concentrations >1 mM [35] and recently a decrease in viability (MTT assay) at 500 mg mL${}^{-1}$ of nanoparticles prepared by reduction of *N*-[3-(trimethoxysilyl)propyl]ethylenediamine [36]. The surface chemistry of these particles was very different to the present alkyl SiQDs (oxide vs alkyl), nevertheless it was important to extend the cytotoxicity studies to higher concentrations and longer duration exposures and in the relevant cell line, CACO-2.

**Figure 5:**
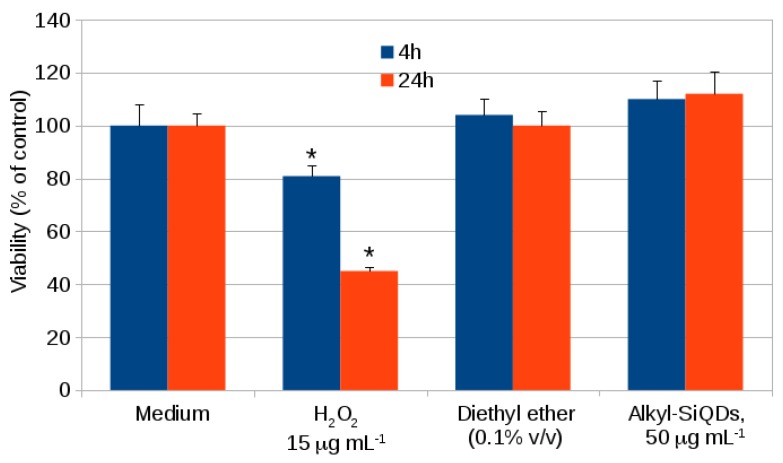
Cytotoxicity studied in CACO-2 after exposure to SiQDs for 4 h and 24 h using the 3-(4,5-dimethylthiazol-2-yl)-2,5-diphenyltetrazolium bromide (MTT) assay. Cell viability (% of control) is plotted as mean ± SD for *n* = 3 experiments. Significant effects (*p* < 0.05) are marked with an asterisk *.

**Figure 6:**
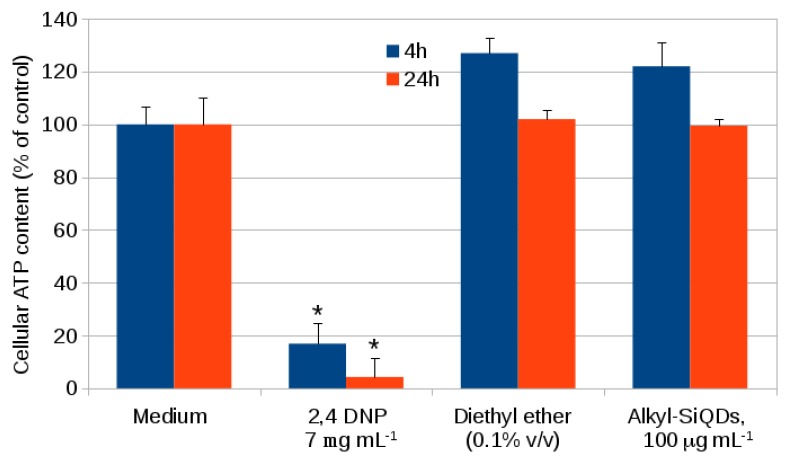
Cellular ATP content measured in CACO-2 cells after exposed to SiQDs for 4 h and 24 h using the fluorescent ATP somatic cell (FLASC) assay (Sigma-Aldrich). The ATP content (% of control) represent with means ± SD for *n* = 3 experiments. Significant effects (*p* < 0.05) are marked with an asterisk *.

Figure 5 shows the results of a standard MTT assay for cell viability for 24 h exposure of CACO-2 to alkyl SiQDs. There is no significant difference between the viability of the CACO-2 in medium, the negative control with vehicle (ether) and the vehicle + alkyl SiQDs at 50 μg mL${}^{-1}$. Hydrogen peroxide at 15 μg mL${}^{-1}$ was used as a positive control to indicate the sensitivity of the assay. The cell viability information from the MTT assay alone cannot classify the level of cytotoxicity and assess the safety of SiQDs. Adverse effects of nanoparticles may also be revealed by the concentration of ATP produced by the cells. High densities and healthy cells generate high concentrations of ATP [37,38]. Data for ATP production using the fluorescent ATP somatic cell (FLASC) assay for similar exposures and up to 100 μg mL${}^{-1}$ alkyl SiQDs are shown in Figure 6. Again, no significant difference between control (medium only), control + vehicle and control + vehicle + SiQDs was observed. The positive control used was 2,4 dinitrophenol (2,4 DNP) which acted as a proton ionophore, disrupted the proton gradient of the mitochondria and inhibited production of ATP. Strong inhibition of ATP production was observed at 7 mg mL${}^{-1}$ of 2,4 DNP.

MTT and ATP production assays were standard, but relatively crude assessments of toxicity and we have also investigated the production of reactive oxygen species (ROS) and DNA damage by single cell electrophoresis (Comet assay). ROS production was measured by the H2DCFDA assay. The 2${}^{\prime }$,7${}^{\prime }$-dichlorodihydrofluorescein diacetate (H2DCFDA) permeated across the cell membrane and the acetate esters were cleaved by intracellular esterases. Oxidation by intracellular ROS then produced a fluorescent species, 2${}^{\prime }$,7${}^{\prime }$-dichlorofluorescein, which may be detected on a standard fluorescence spectrometer.

**Figure 7:**
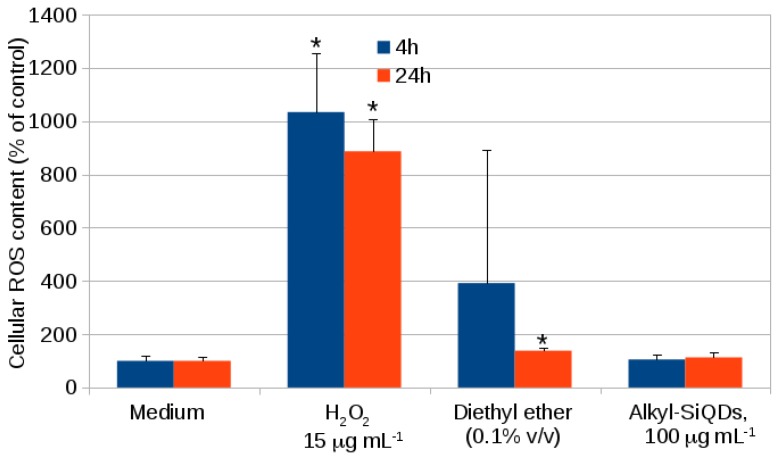
Cellular ROS content measured in CACO-2 cells after exposure to SiQDs for 4 h and 24 h using H2DCFDA (2${}^{\prime }$,7${}^{\prime }$-dichlorodihydrofluorescein diacetate, Sigma-Aldrich, Gillingham, UK). The ROS contents (% of control) are indicated as means ± SD for *n* = 3 experiments. Significant effects (*p* < 0.05) are marked with an asterisk *.

Figure 7 shows the data obtained for exposures of 4 h and 24 h with the same positive and negative controls as in the cell viability data of Figure 5. Some evidence of ROS production due to ether was observed, but this may be an artefact due to the light sensitivity of H2DCFDA. It is also noteworthy that no significant difference was observed between the control (medium alone) and the alkyl SiQDs in medium + vehicle. Finally, 15 μg mL${}^{-1}$ of hydrogen peroxide produced an approximately ten-fold increase in ROS and this effect may be responsible for the decrease of cell viability in the positive control of Figure 5.

**Figure 8:**
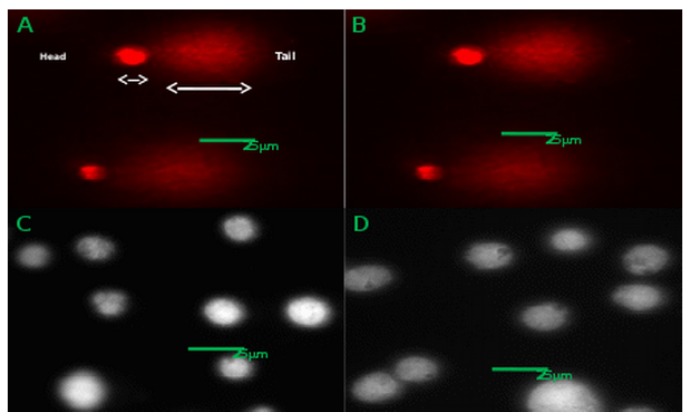
(**A**,**B**) Comet images of CACO-2 cells after exposure to H${}_{2}$O${}_{2}$ (15 μg mL${}^{-1}$) and (**C**,**D**) Images of CACO-2 cells after exposure to SiQDs (200 μg mL${}^{-1}$) for 24 h using Comet IV (Perceptive, Bury St Edmunds, UK).

Figure 8 is representative of the DNA damage observed on exposure of CACO-2 to hydrogen peroxide and alkyl SiQDs. Panels (A, B) of Figure 8 illustrate the DNA damage caused by hydrogen peroxide in the positive control. The typical comet-shape was observed, where the diffuse ‘comet tail’ represents that part of the cellular DNA that has undergone strand breakage and migrates more rapidly towards the anode. In contrast, panels (C, D) of Figure 8 show typical intact comet ‘heads’ in the presence of 200 μg mL${}^{-1}$ alkyl SiQDs for 24 h; these images indicate the lack of DNA damage due to SiQDs.

**Figure 9:**
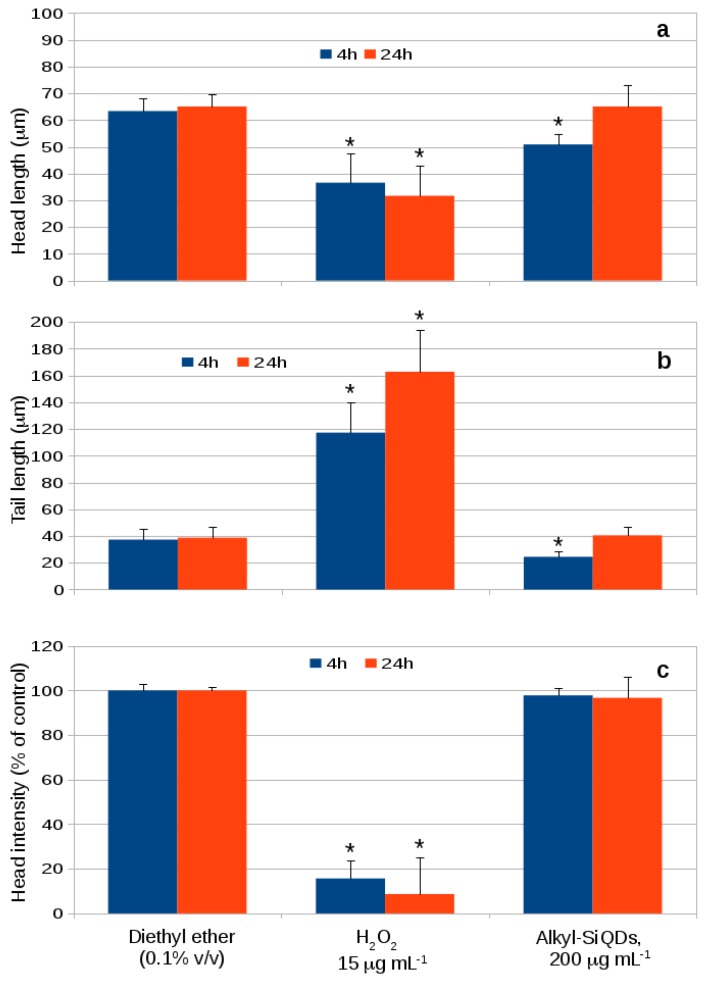
DNA damage in CACO-2 cells after exposed to SiQDs (200 μg mL${}^{-1}$) for 4 h and 24 h determined by Comet IV (Perceptive, UK). Head length (**a**), tail length (**b**) and head intensity (**c**) are presented as means ± SD. Significant effects (*p* < 0.05) are marked with an asterisk *.

The comet assay is quantified in terms of head and tail lengths and head intensities in Figure 9. A small difference was observed between head length in the control and exposure to alkyl SiQDs at 200 μg mL${}^{-1}$ for 4 h in Figure 9a. However, no significant difference is observed after 24 h. The positive control, 15 μg mL${}^{-1}$ hydrogen peroxide, gives a small reduction in head length, but a large increase (three-fold) in tail length (Figure 9b). The clearest distinction between the samples was in head intensity (Figure 9c) where an order of magnitude decrease was observed in the positive control and no significant difference for alkyl SiQDs.

**Figure 10:**
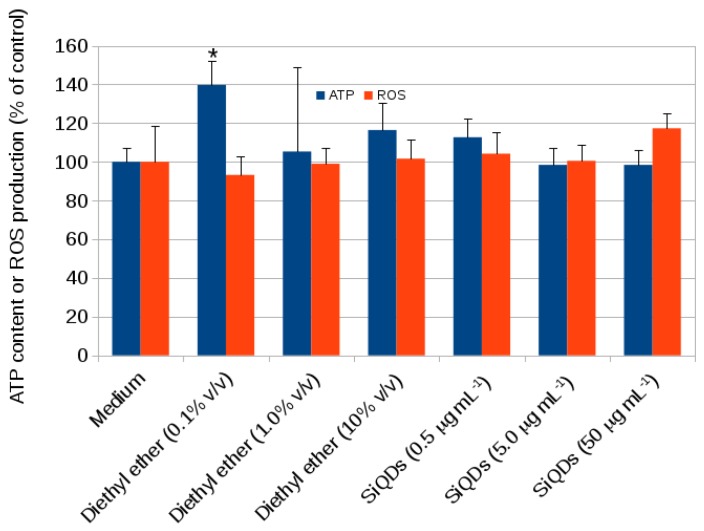
Cellular ATP content measured in CACO-2 cells after exposure to SiQDs for 14 days using FLASC (Sigma-Aldrich). The ATP contents (% of control) are indicated as means ± SD for *n* = 3 experiments. Cellular ROS content measured in CACO-2 cells after exposed to SiQDs for 14 days using H2DCFDA (Sigma-Aldrich). The ROS content (% of control) is indicated as means ± SD for *n* = 3 experiments. Significant effects (*p* < 0.05) are marked with an asterisk *.

Finally, Figure 10 shows an assessment of cytotoxicity of alkyl SiQDs at concentrations up to 50 μg mL${}^{-1}$ for 14 days of exposure. Neither ATP production (FLASC assay) nor ROS production (H2DCFDA assay) were significantly affected and the data also indicated that the vehicle (diethyl ether), used to dispersed the alkyl SiQDs in the culture medium, had no effect on these parameters.

Nanoparticles are hypothesized to generate cytotoxicity, oxidative stress, and inflammatory responses [39,40,41]. However, in this study neither cytotoxicity nor increased oxidative stress was detected in CACO-2 cells treated with alkyl-SiQDs. This data is consistent with earlier studies on HeLa [20] and HepG2 [33]. In summary, alkyl-SiQDs are readily internalized by CACO-2 as previously observed for other cell lines [20]. They showed no evidence of cytotoxicity for concentrations up to 200 μg mL${}^{-1}$ and exposures of duration up to 14 days.

### 2.2. Endocytosis of Alkyl SiQDs

After establishing the lack of acute or chronic toxicity of alkyl-SiQDs in CACO-2 cells, we investigated aspects of the mechanism of endocytosis. As part of the study, we broadened the range of cell lines to include HeLa, HepG2 and Huh7. Huh7 and HepG2 are liver cancer cell lines and useful for the study of liver metabolism and the toxicity of xenobiotics.

Figure 1 illustrates schematically the clathrin-mediated and caveolae-mediated endocytosis mechanisms. The clathrin-mediated process involves self-assembly of the clathrins to create a coated pit in the membrane and subsequent formation of clathrin-coated vesicles of diameter about 100 nm [42,43]. Caveolin dimeric protein binds with cholesterol and coats the surface of membrane invaginations to form caveolin coated pits [44]. The caveolin coated pits are also known as plasma membrane lipid rafts [45]. Although the functions of clathrin and caveolin coated pits are similar; they transport macromolecules and are implicated in a wide range of cell physiology functions [46], cholesterol is a key component to distinguish hlcaveolin- from clathrin-coated pits [45]. The cholesterol sensitivity of caveolae can also be used to distinguish the caveolin-mediated pathway from other mechanisms such as clathrin-dependent and clathrin-caveolin-independent endocytosis [47,48]. The size of caveolae was about 100 nm diameter and 100 nm depth [49]. There was evidence from inhibitor and co-localization studies with latex beads that particles of diameter <200 nm are internalized via the clathrin-coated pits, but at larger sizes the caveolae-mediated pathway is predominant and the particles are no longer delivered to the lysosome [50]. However in the case of very small, but hydrophobic nanoparticles, such as alkyl-SiQDs of diameter 5 nm, it is not clear which pathway is favoured.

**Figure 11:**
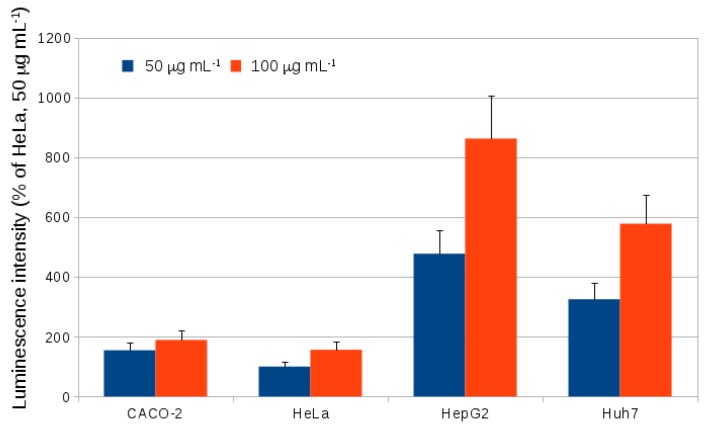
Luminescent intensities in excess of the background from the control (HeLa, no SiQDs present) measured using flow cytometry (excitation 355 nm, emission 675 nm) for cells treated with 50 or 100 μg mL${}^{-1}$ SiQD for 24 h. The values reported are normalized to the intensity for HeLa treated with 50 μg mL${}^{-1}$ SiQD for 24 h. Bars are means ± SD for *n* = 3 experiments.

In previous work, evidence for the cholesterol-dependence of endocytosis of alkyl SiQDs by HeLa cells was obtained using MβCD and filipin as inhibitors of cholesterol-dependent endocytosis and a confocal microscope to assess inhibition of endocytosis [20]. This supports the involvement of lipid rafts and a caveolin mediated pathway for endocytosis in these cells. However, it must be noted that such inhibitors are very toxic [51] and therefore we employed a different strategy in this paper to address the mechanistic question. A range of cell lines (CACO-2, HeLa, HepG2, Huh7) were chosen and the extent of internalization of alkyl-SiQDs was quantified by flow cytometry; we then used gene expression analysis to correlate these findings with the amount of caveolin proteins in each cell line. The present work confirmed by flow cytometry that HeLa cells can accumulate SiQDs (Figure 11). The liver tumour cell lines, HepG2 and HuH7, showed significantly higher levels of accumulation compared with HeLa, but CACO-2 showed levels of SiQD internalization similar to HeLa. It is not clear why these cells should accumulate SiQDs more than HeLa (cervical tumour) or CACO-2 (colon tumour).

### 2.3. Caveolin Gene Expression

**Figure 12:**
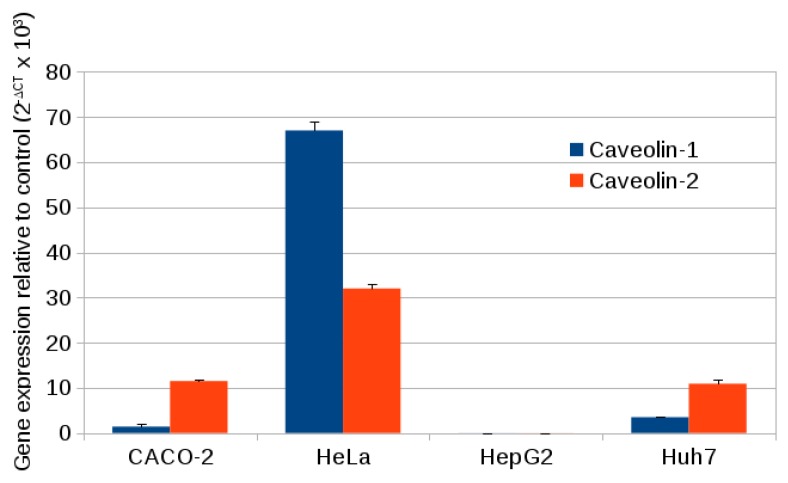
Caveolin1 and caveolin2 expression values compared with the control gene (glyceraldehyde-3-phosphatedehydrogenase (GAPDH)) in CACO-2, HeLa, HepG2, and Huh7 (*n* = 3 experiments).

Caveolin-1 and caveolin-2 gene expression was quantified using quantitative PCR (qPCR). RNA was extracted from the cells and cDNA strands were synthesized using Moloney Murine Leukemia Virus (M-MuLV) reverse transcriptase. Quantitative PCR of the DNA used a fluorescent reporter and the intensity of this fluorescence was monitored against the number of PCR cycles. The control gene was that for glyceraldehyde-3-phosphatedehydrogenase (GAPDH). The data are reported in Table 1. CT is the number of PCR cycles required to amplify the reporter to a threshold and therefore the amount of cDNA in the sample is proportional to $2^{CT}$. The amount of the control (GAPDH) was constant amongst the four cell lines chosen, but there are significant variations in the amounts of the two caveolins. Figure 12 shows the expression of two caveolins (caveolin-1 and caveolin-2) relative to the control gene. Hela expressed both caveolins; caveolin1 at a high level and caveolin-2 at a moderate level. Caveolin expression in the other cell lines was lower overall. In line with other reports [52,53] there was very low expression of both isoforms in HepG2 cells [54]. Huh7 and CACO-2 both showed much lower expression of caveolin-1 than in HeLa, although caveolin-2 expression was higher than caveolin-1. In comparison of the expression data to the internalization data of Figure 11, some conclusions can be reached concerning the mechanism of endocytosis of alkyl SiQDs in the four cell lines. First, the mechanism is unlikely to involve caveolae in the HepG2 cells because they were the most active for the internalization of alkyl SiQDs, but expressed almost undetectable levels of caveolins. Second, the high levels of expression of caveolin in HeLa coupled with previous observations of cholesterol-dependence are consistent with caveolin-mediated endocytosis.

### 2.4. Chlorpromazine Inhibition

The clathrin-mediated endocytosis inhibitor, chlorpromazine, acted on the plasma membrane to inhibit coated pit formation [55]. We therefore attempted to observe inhibition of alkyl-SiQD uptake to provide evidence for a contribution of the clathrin-mediated pathway. The cells were pretreated with 30 μM chlorpromazine for 1 h and then incubated with 100 μg mL${}^{-1}$ alkyl-SiQDs and chlorpromazine for 24 h. Chlorpromazine is very toxic and effects on cell morphology were apparent—the HeLa and Huh7 cells no longer elongated and showed a circular morphology. Because of the effect of chlorpromazine on cell morphology, data obtained using this inhibitor needs to be treated with caution [51]. However, there was no evidence for an inhibitory effect on uptake of alkyl-SiQDs. Even a small increase in uptake of alkyl-SiQDs was observed for Huh7 cells (Table 2). Such an effect has been reported previously for the uptake of lactosylceramide, which is thought to occur via a clathrin-independent mechanism, in both ARPE-19 and Huh7 cells [51].

## 3. Materials and Methods

### 3.1. Preparation of Alkyl-SiQDs and Dispersions in Aqueous Media

Unless otherwise indicated, all reagents were obtained from Sigma-Aldrich, Gillingham, UK. Preparation of ${}^{n}C_{11}$-capped silicon quantum dots (alkyl SiQDs). SiQDs were prepared according to established procedures [20]. The wafer, p-Si<100> (10 Ω cm resistivity, Compart Technology, Peterborough, UK), was cut into 1.1 × 1.1 = 1.2 cm${}^{2}$ chips. Each chip was anodically etched in a 1:1 *v*/*v* solution of 48% aqueous HF and ethanol (95% Fisher Scientific) to produce porous silicon at a current density of 500 mA cm${}^{-2}$ for 5 min. The porous silicon formed a roughly circular brown-yellow area with intense orange fluorescence under a handheld Hg lamp (Figure 2C,D). Next, the porous silicon chips (×4) were placed in a Schlenk flask and refluxed under dry N${}_{2}$ in an anhydrous toluene solution (20 mL) containing 1 M of undec-1-ene. During the reflux, hydrosilation occurred at the hydrogen-terminated Si surface and the stress of bubble formation broke the porous layer into individual SiQDs. The SiQDs were produced as a lyophillic sol in toluene and may be evaporated to dryness as a yellow wax that fluoresces orange under an Hg lamp (Figure 2A,B). The mass of SiQDs formed per chip was of the order of 200 μg.

The alkyl-SiQDs are soluble in nonpolar solvents and a lyophobic dispersion was prepared by injection of small volumes of an ether solution of the SiQDs into aqueous media [16]. Diethyl ether was the solvent of choice for this process because it evaporates quickly and leaves a suspension of SiQDs, however we also controlled for the possible effects of ether residues in our assays by injection of ether into medium without SiQDs.

### 3.2. CACO-2 Cell Culture

Sterile plastic syringes, cryo-tubes and pipettes were obtained from Fisher Scientific hlHampton, NH, USA. Unless otherwise indicated, all cell culture reagents were obtained from Sigma-Aldrich, Gillingham, UK and the product numbers are indicated below. CACO-2 cells (European Collection of Cell Cultures (ECACC) Public Health England, UK) were cultured in 75 cm${}^{2}$ flasks (Greiner, filter cap, Sigma-Aldrich, UK) in sterile complete medium (syringe filter pore size 0.22 μm, diam. 33 mm, Sigma-Aldrich, UK) containing 15 mL of Dulbecco’s modified Eagle’s medium (D1145). For fluorescence imaging experiments a phenol red-free DMEM was used. The medium was supplemented with 20% (*v*/*v*) foetal Bovine serum (FBS) (F9665), 1% (*v*/*v*) penicillin/streptomycin (P0781), 1% (*v*/*v*) MEM non-essential amino acid solution (M7145), 1% (*v*/*v*) l-glutamine solution (G7531), and 1% (*v*/*v*) sodium pyruvate solution (S8636). The cells were grown at 37 ${}^{\circ }$C in 5% CO${}_{2}$. The cell culture medium was changed every 2–3 days by replacing with warmed Dulbecco’s phosphate buffered saline (PBS) (D8537) and after washing twice, warmed complete medium was added using a sterile 10 mL pipette. After reaching 70–80% confluence, cells were sub-cultured using trypsin-EDTA solution (5× dilution) (59418C). The culture medium was replaced with warmed PBS and washed twice. Then 5 mL of trypsin was added to replace the PBS and left on the cells for approximately 5 min till the cells were dissociated. Warmed complete medium (10 mL) was then added to inhibit the trypsin reaction. The cell suspension was transferred to a 50 mL centrifuge tube (Fisherbrand, Fisher Scientific) and a cell pellet was collected by centrifugation at 1000× *g* for 5 min. This pellet was used for new cultures or experiments or for cell storage. For new cultures/experiments, the cell pellet was re-suspended in warmed complete medium and divided into portions. For new cultures, the cell solution was pipetted into a new flask and made up to 10 mL with fresh warmed medium. Cell stocks were stored in liquid nitrogen for further studies. To freeze down cells, the cell pellet was re-suspended in PBS and centrifuged at 1000× *g* for 5 min. The cell pellet was re-suspended in 10% (*v*/*v*) (dimethyl sulfoxide (DMSO) in FBS and aliquoted (1 mL) into cryo-tubes. The tubes were frozen slowly and stored at −80 ${}^{\circ }$C overnight. After 24 h the tubes were transferred to liquid nitrogen for long-term storage.

To grow the cells, a cryo-tube was thawed at room temperature. After thawing, the cell solution was transferred into a 15 mL centrifuge tube (Fisherbrand, Fisher Scientific) and mixed with 4 mL warmed complete medium. The cell pellet was collected by centrifugation at 1000× *g* for 5 min and re-suspended in 10 mL warmed complete medium. The cell solution was placed in a 75 cm${}^{2}$ flask and incubated at 37 ${}^{\circ }$C with 5% CO${}_{2}$.

### 3.3. Optical and Epifluorescence Microscopy

Fluorescence images were obtained using an epifluorescence microscope (Axioskop 2, Zeiss, Jena, Germany) using AxioVision version 4.8 software (Zeiss). The excitation source was an Hg arc lamp and was bandpass filtered to give light of wavelength 300 < λ < 400 nm. Back-scattered light from the sample was longpass filtered λ > 420 nm to remove elastically scattered light and the luminescence was captured on a monochrome CCD camera (AxioCam HRm, Zeiss). The integration time for the CCD was automatically adjusted by the software to maximize the detected signal. The same microscope was used to capture bright field optical images for cell morphology studies, with white light supplied by a halogen bulb. The software ImageJ (https://imagej.nih.gov/ij/, version 1.49) was used to superimpose the fluorescence images on the optical images. A red-orange false color, that approximately represents the actual SiQD fluorescence spectrum, was used to represent the fluorescence detected by the monochrome camera.

### 3.4. Observation of Internalization of SiQDs by Epifluorescence Microscopy

CACO-2 ($5\times 10^{3}$ cells mL${}^{-1}$) were cultured on cleaned coverslips (Scientific Laboratory Supplies Ltd., Nottingham, UK) overnight. After 24 h, the cells were treated with freshly prepared 0.2 μg mL${}^{-1}$ SiQDs from a stock solution of 1 mg mL${}^{-1}$ SiQDs in phenol red-free medium for 4 h. The treated cells were washed three times with phosphate-buffered saline (PBS). To visualize SiQDs inside the living cells, the coverslip was taken out from the plate and placed on the top of the slide with the cells on the top side. Then the coverslip was sealed with 50 μL Mowiol mounting medium and another clean coverslip.

To visualize the optical images of cells after 14 days ‘chronic’ exposure, 5% (*v*/*v*) of 70–80% confluence of CACO-2 ($10^{3}$ cells mL${}^{-1}$) were cultured flasks (filter cap), treated surface area 25 cm${}^{2}$ (T25), overnight. After 24 h, the cells were treated with various concentrations of freshly preparation SiQDs at 0.5, 5, and 50 μg mL${}^{-1}$ from a stock solution of 1 mg mL${}^{-1}$ SiQDs in 10 μL diethyl ether (ether) in phenol red-free medium. The cells were treated with SiQDs for 14 days and the medium were replaced with a fresh preparation of SiQDs suspension every two days. After 14 days the cells were washed with PBS three times. Then the cells were removed from the flasks using trypsin. The cell pellets were then collected and suspended in 500 μL PBS. To visualize SiQDs inside the cells, 50 μL of the cell suspension was mixed with 50 μL Mowiol mounting medium and 50 μL of the mixture dropped on the top of a clean slide. The slide then was covered with clean coverslips.

### 3.5. CACO-2 Cell Viability (MTT Assay)

CACO-2 cells (1–2 × 10${}^{5}$ cells mL${}^{-1}$) were cultured overnight in 96 well and 12 well plates. The cells were then treated with 50 μg mL${}^{-1}$ of freshly preparation SiQDs, positive control (15 μg mL${}^{-1}$ H${}_{2}$O${}_{2}$), and controls (untreated and ether-treated) for 4 h and 24 h. The treated cells were washed and incubated with 3-(4,5-dimethylthaiazol-2-yl)-2,5-diphenyltetrazolium (MTT, 5 μg mL${}^{-1}$). After 2 h incubation, the MTT solution was removed and the dark purple color from the cells was dissolved in neat isopropanol and allowed to stand for a further 10 min. Finally, the solution (0.1 mL) was removed from the 12 well plates and transferred to 96 well plates. The absorbance at 570 nm was measured using a spectrophotometer (Thermo Scientific Multiskan microplate reader (96 wells plate)). The percentage cell viability was calculated and compared with the control cells. Results are reported as the mean ± SD of triplicate independent experiments.

### 3.6. CACO-2 Intracellular ATP Content (FLASC Assay)

An adenosine 5-triphosphate (ATP) bioluminescent somatic cell assay kit (Sigma-Aldrich) was used to measure cellular ATP content. CACO-2 cells were incubated with SiQDs at 100 μg mL${}^{-1}$ in culture medium for 4 h and 24 h or with SiQDs at 0.5, 5, or 50 μg mL${}^{-1}$ for 14 days. As a positive control, cells were also treated with 2,4 DNP (7 mg mL${}^{-1}$) for 4 h and 24 h. ATP was extracted from CACO-2 cells (50 μL) using somatic cell ATP releasing reagent (FLSAR (100 μL). The extract was decanted in to micro UV cuvettes containing luciferin-luciferase solution and MgSO4 (FLAAM, FLAAB). The bioluminescence ($\lambda_{max}=560$ nm) was observed in a fluorescence spectrometer (SpexFluoroMax/GRAMS 32, Horiba, Kyoto, Japan) with the instrument’s excitation beam blocked with a piece of black card. Results were reported as the mean ± SD of triplicate independent experiments.

### 3.7. CACO-2 Oxidative Stress (H2DCFDA Assay)

CACO-2 cells were incubated with either SiQDs at 100 μg mL${}^{-1}$ for 4 h and 24 h or with SiQDs at 0.5, 5 or 50 μg mL${}^{-1}$ for 14 days. The cells were then incubated with 0.5 mg mL${}^{-1}$ H2DCFDA (2${}^{\prime }$,7${}^{\prime }$-dichlorodihydrofluorescein diacetate, stock 20 mM in DMSO) in culture medium for 45 min. The H2DCFDA cell solutions were transferred to micro UV cuvettes and the fluorescence was measured immediately at an excitation wavelength of 485 nm and an emission wavelength at 530 nm (SpexFluoroMax/GRAMS 32 fluorescence spectrometer).

### 3.8. CACO-2 DNA Damage (Comet Assay)

CACO-2 cells treated with SiQDs (200 μg mL${}^{-1}$) for 4 h and 24 h, positive controls (cells treated with 15 μg mL${}^{-1}$ H${}_{2}$O${}_{2}$) and control (diethyl ether-treated) cells were evaluated for DNA damage using single cell gel electrophoresis (SCG) (Comet assay) by an established method (Singh 1988). The alkaline Comet assay (pH > 13) measures DNA lesions including single and double DNA breaks and alkali-labile sites (Singh et al., 1988, Tice et al., 2000). The CACO-2 cells were cultured in a T-25 flask at a density of $5\times 10^{5}$ cells cm${}^{-2}$ in 4 mL DMEM for 24 h before treatment. After treatment, the cells were gently harvested using trypsin and the cell pellet was re-suspended in 1 mL of cool PBS in amber microcentrifuge tubes and stored on ice. In the dark room under dim light, a mixture of equal parts cell suspension and 1% (*w*/*v*) low melting agarose (Flowgen Bioscience) in PBS (37 ${}^{\circ }$C) was prepared (200 μL in total). The mixed solutions (60 μL) were dropped on slides pre-coated with low melting agarose, cover slips were placed on top and the mixture was allowed to solidify on ice. After removing the coverslips, the slides were gently immersed in chilled lysing solution (2.5 M NaCl, 100 mM EDTA, 10 mM Tris, Triton X-100 (1% *v*/*v*), DMSO (1% *v*/*v*)). The slides were then transferred into chilled alkaline buffer (300 mM NaOH, 1 mM EDTA, pH > 13) in an electrophoresis tank and left for 30 min to allow DNA unwinding. Electrophoresis was performed at a constant voltage of 22 V, with a current of 0.5–0.7 mA for 30 min in the same alkaline buffer. The slides were then placed into chilled neutralising buffer solution (0.5 M Trisma base pH 7.5) to allow the DNA to reconstitute. Finally, the DNA slides were dried and stained with SYBR gold (Life Technologies) as a solution (1:10,000 (*v*/*v*) in 1X Tris-EDTA (0.1 M Trisma base, 0.05 M EDTA). The following day, the cells were re-hydrated with water and fluorescent cells images, ×200 magnification (Olympus U-RFLT50/Olympus CX40, Southend-on-Sea, UK) with excitation wavelength 490 nm were counted (50 images for each slide and 2 slides for each treatment). As the glowing cells were counted, the counting capture images were directly connected to the image analysis program (Comet Assay IV, Instem) and the following parameters recorded: head length, tail length, and head intensity of DNA. The independent Comet experiments were performed in duplicate. Results are the mean ± SD of two whole cell samples (100 cells) in two independent experiments.

### 3.9. CACO-2 Chronic Exposure to Alkyl SiQDs

For studies of chronic exposure, CACO-2 ($10^{3}$ cells mL${}^{-1}$) were cultured in T25 culture flasks overnight. After 24 h, the cells were treated with various concentrations of freshly prepared SiQDs at 0.5, 5, or 50 μg mL${}^{-1}$ from a stock solution of 1 mg mL${}^{-1}$ SiQDs in 10 μL diethyl ether (ether) in phenol red-free medium. The cells were treated with SiQDs for 14 days and the medium was replaced with a fresh preparation of SiQDs suspension every two days. After 14 days the cells were washed with PBS three times and detached using trypsin solution. The cell pellets were then suspended in 500 μL PBS. ATP and ROS production were measured using the fluorescent ATP somatic cell (FLASC) and 2${}^{\prime }$,7${}^{\prime }$-dichlorodihydrofluorescein diacetate (H2DCFDA) assays.

### 3.10. Internalization of SiQDs in Different Cell Lines

CACO-2, Huh7, Hela, and HepG2 were seeded in separate T-25 flasks (Sigma-Aldrich, UK) at a density of $1.5\times 10^{5}$ cells mL${}^{-1}$ in the appropriate medium and incubated at 37 ${}^{\circ }$C with 5% CO${}_{2}$. CACO-2 cells (European Collection of Cell Cultures (ECACC) Public Health England, UK) were cultured as described above. Hela, HepG2, and Huh7 cells (Institute of Cellular Medicine, Newcastle University) were cultured in complete RPMI-1640 medium (R7509, Sigma-Aldrich, UK) with 10% (*v*/*v*) FBS instead of Dulbecco’s modified Eagle’s medium. However, all other cell culture procedures were performed exactly as described for CACO-2.

After 24 h the cell medium was replaced with complete medium containing 0.1% (*v*/*v*) foetal bovine serum (FBS) and freshly prepared 50 or 100 μg mL${}^{-1}$ SiQDs in 0.5% (*v*/*v*) diethyl ether and incubated at 37 ${}^{\circ }$C with 5% CO${}_{2}$ for 24 h. In a separate flask, the cells were incubated in the complete medium containing 0.1% (*v*/*v*) FBS with no SiQDs but the equivalent volume of diethyl ether. These were used as the control flasks. After 24 h the culture medium was replaced with PBS and washed three times to eliminate the treated samples. Then the PBS was replaced with trypsin and left for 3 min. The trypsin reaction was stopped by adding complete medium containing 10% (*v*/*v*) FBS. To remove FBS the cell suspension was washed in PBS using a cell washer (BD FACS^TM^ Lyse/Wash Assistant, Franklin Lakes, NJ, USA) with precipitation force 461 g, precipitation time 10 s, wash force 350 g, and wash volume 6400 μL. The precipitated cell suspensions were used for measuring luminescent intensities inside the cells.

To compare luminescence intensity of SiQDs inside the cells among various cell types, the luminescence intensities were recorded in each individual cell. In each sample 3000 events were collected using a BD LSRII flow cytometer. In the BD LSRII system, several excitation and detection wavelengths are available; SiQDs show strong orange-red luminescence, but have a large Stokes shift and are best detected with low excitation wavelength (355 nm) and at a long detection wavelength (675 nm). The data are presented as average luminescence intensities (Mean ± SD).

### 3.11. Specific Gene Expression of Caveolin 1 and Caveolin 2

#### 3.11.1. RNA Preparation

RNA was extracted from cells at 70–80% confluence. The cells were washed three times with PBS and then treated with trypsin. To produce a cell pellet, the trypsin reaction was stopped using cell culture medium containing 10–20% (*v*/*v*) FBS and centrifuged at 1000 *g* for 5 min. The cell pellet was re-suspended in PBS and centrifuged at 1000 *g* for a further 5 min to obtain a clean cell pellet for RNA extraction. Tri-reagent (Sigma-Aldrich) (1 mL) was added to the cell pellet and after mixing, the cell suspension was transferred into an ribonuclease (RNase)-free screw cap 1.5 mL micro centrifuge tube and incubated at room temperature for 10 min to allow the reagent to dissociate the nucleoprotein complexes. Next, 100 μL of 1-bromo-3-chloropropane was added and mixed until the suspension solution became a milky pink color; it was then incubated at room temperature for another 10 min. Then suspension was centrifuged at 12,000× *g*, 4 ${}^{\circ }$C for 10 min. After centrifugation, the cell suspension revealed three different layers. The first layer on the top was a clear RNA-containing solution, the middle, opaque layer contained the DNA and the third layer in the bottom was the dark pink solution comprising mainly protein. The first layer (500 μL) was collected and placed into a new RNase-free micro centrifuge tube. To precipitate the RNA, 500 μL of isopropanol was added to the tube, mixed well by vortex and incubated at room temperature for 10 min. RNA was separated by centrifugation at 12,000× *g*, 4 ${}^{\circ }$C for 10 min. The isopropanol was carefully decanted and the white RNA pellet was collected. The pellet was re-suspended in 75% (*v*/*v*) ethanol in RNase-free water and vortexed till the pellet moved out from the bottom of the tube. To collect the RNA, the suspension was centrifuged at 7500× *g*, 4 ${}^{\circ }$C for 5 min. The ethanol was decanted and the RNA pellet dried at room temperature for 10 min. The RNA pellet was re-suspended in 50 μL RNase-free water and mixed well by vortex. The RNA solution was stored at −80 ${}^{\circ }$C and checked for purity using a Nanodrop spectrophotometer ND-1000 (Thermo Scientific, Waltham, MA, USA) before performing cDNA synthesis. An RNA solution absorbance of 1 at a wavelength of 260 nm is equivalent to 40 μg mL${}^{-1}$ RNA. The purity of the RNA was assessed using the ratio of RNA solution absorbance at 260 nm to 280 nm. All preparations showed A260:A280 ratios between 1.95 and 2.0 indicating sufficient purity from contamination by protein.

#### 3.11.2. cDNA Synthesis

The cDNA strand was synthesized using M-MuLV Reverse Transcriptase (200,000 U mL${}^{-1}$) (New England Biolabs, Hitchin, UK) with random oligonucleotide sequences as primers (Random Hexamers (0.4 μg μL${}^{-1}$)), dNTPs solution mix (10 mM in each of dATP, dCTP, dGTP, and dTTP), RNase-free water, 10× Reverse transcriptase Reaction Buffer (1X: 75 mM KCl, 50 mM Tris-HCl, 3 mM MgCl${}_{2}$, 10 mM dithiothreitol, pH 8.3) and RNase Inhibitor (40,000 U mL${}^{-1}$). First, 1 μL of random hexamers, 1 μL of dNTPs and 6 μL of RNase-free water were added to an RNase-free micro centrifuge tube and placed on ice (solution (1)). A second solution (2) was prepared by adding 2 μL 10× buffer, 0.1 μL RNase inhibitor, 0.25 μL reverse transcriptase enzyme, and 7.65 μL RNase-free water to another RNase-free micro centrifuge tube and placed on ice. Then 2 μL of the RNA solution was added to (1), mixed well and incubated at 65 ${}^{\circ }$C for 10 min. The denatured RNA solution was cooled on ice for 2 min to allow the primers to recombine with the RNA. Then solution (2) was added and mixed well. The combined solution was incubated at 37 ${}^{\circ }$C for 50 min to allow the reverse transcription of RNA to cDNA. To inactivate any remaining enzyme, the cDNA solution tube was incubated at 70 ${}^{\circ }$C for 15 min. The cDNA solution was stored at −20 ${}^{\circ }$C prior to qPCR analysis.

#### 3.11.3. Quantitative Reverse Transcriptase Real Time PCR Analysis

Gene expression assays, TaqMan^TM^ caveolin 1 and caveolin 2 (Life Technologies (Thermo Fisher Scientific), Waltham, MA, USA), were employed to quantify specific gene expression in various cells types compared with the control gene, glyceraldehyde-3-phosphate dehydrogenase (GAPDH). The amplified caveolin 1 or 2 gene with included FAM^TM^ reporter dyes and control gene with VIC^TM^ reporter dye were recorded by qPCR using a MicroAmp^TM^ Fast Optical 48-well PCR plate (Applied Biosystems, Foster City, CA, USA) on an Applied Biosystems StepOne^TM^ Real-Time PCR system. Gene expression levels are reported as the number of amplification cycles, CT, required to exceed a set fluorescence threshold determined by the baseline (control gene) in the exponential phase of amplification. A low CT represents a large amount of gene expression [56].

### 3.12. Statistical Analysis

Statistical analysis was performed using SPSS software (IBM Statistics 21). The level of significance for the effects and interactions of parameters were calculated using one-way ANOVA. The results are presented as the means and standard deviations (Mean ± SD). Post Hoc tests were used for the comparison for the significant parameters. A *p* value ≤ 0.05 was taken as statistically significant.

## 4. Conclusions

Alkyl-capped SiQDs were internalized by CACO-2 without observable toxic effects for the longest durations of exposure studied (14 days). No acute effect of alkyl SiQDs on cell viability (MTT assay) or DNA damage (Comet assay) was observed after 24 h exposure at concentrations up to 100 μg mL${}^{-1}$ (MTT) or 200 μg mL${}^{-1}$ (Comet). No chronic toxicity was observed either after 14 days exposure to 50 μg mL${}^{-1}$ alkyl SiQDs as judged by microscopic observation of cell morphology, ROS production (H2DCFDA assay) or ATP production (FLASC assay). The extent of uptake of alkyl SiQDs varies between different cell lines. Flow cytometry indicated that CACO-2 and HeLa cells internalized alkyl SiQDs to a similar extent after 24 h exposure to 50 or 100 μg mL${}^{-1}$. However, under the same conditions, the liver cancer cell lines HepG2 and Huh7 showed significantly greater extents of internalization, by a factor of about 5 in the case of HepG2. Previous work indicated a cholesterol-dependence of the endocytosis of alkyl SiQDs by HeLa cells which suggested the involvement of lipid rafts and caveolin-mediated endocytosis. No inhibitory effect of the clathrin-mediated endocytosis inhibitor, chlorpromazine was observed, although it is difficult to reach firm conclusions because of the toxicity of this agent. Gene expression analysis revealed wide variations in the expression of caveolins 1 and 2 by the four cells lines, with similar levels in CACO-2 and Huh7, but much larger levels in HeLa and very little in the most active cell line, HepG2. It is therefore likely that the mechanism of endocytosis of SiQDs is cell-line dependent and may involve more than one pathway in any given cell. 

## Figures and Tables

**Table 1:** Gene expression given as the number of quantitative pCR (qPCR) cycles required to reach a threshold level, $C_{T}$. The values reported are the mean and standard deviation obtained from *n* = 3 experiments. ΔCT values derived from this data are plotted in Figure 12.

Cell Line	Caveolin-1	GAPDH	Caveolin-2	GAPDH
CACO-2	27.03 ± 0.06	17.51 ± 0.02	23.99 ± 0.07	17.55 ± 0.03
HeLa	21.15 ± 0.08	17.24 ± 0.04	2.32 ± 0.06	17.33 ± 0.02
HepG2	35.69 ± 0.31	17.65 ± 0.02	34.89 ± 0.43	17.66 ± 0.03
Huh7	25.92 ± 0.05	17.77 ± 0.02	24.33 ± 0.17	17.81 ± 0.02

**Table 2:** Inhibition of alkyl-SiQD uptake after exposure to the clathrin-mediated endocytosis inhibitor chlorpromazine. The cells were pre-treated with 30 μM chlorpromazine for 1 h and then 100 μg mL${}^{-1}$ alkyl-SiQDs and 30 μM chlorpromazine for 24 h. The values presented are fluorescence intensities determined by flow cytometry as percentage of the control in the absence of inhibitor. (means ± SD for n=3 experiments).

Cell Line	Intensity as % of Control
HeLa	94 ± 6.9
Huh7	170 ± 26
